# On the Constitution and Physiology of the Bile

**Published:** 1857-11

**Authors:** 


### ECLECTIC DEPARTMENT,

### AND SPIRIT OF THE MEDICAL PERIODICAL PRESS

---

*On the Constitution and Physiology of the Bile.*—By JNO: C. DALTON, Jr., M. D., Professor of Physiology and Microscopic Anatomy in the College of Physicians and Surgeons, New York.

Notwithstanding the readiness with which the bile may be obtained for purposes of examination, the evident importance of the secretion, and the labor which has been bestowed upon it, it must be confessed that we are still very far from having a complete idea of its nature and function as one of the intestinal fluids. The present condition of our knowledge in regard to it, may be briefly summed up as follows: Since the analyses of Strecker, published in 1848 and '49, it has been known that the bile contains, as its essential and characteristic ingredients, two saline substances, the glykocholate and the taurocholate of soda; and that the organic acids of these salts, glykocholic and taurocholic acid, both contain nitrogen; and the latter, in addition to it, two equivalents of sulphur. Besides these peculiar or characteristic ingredients, the bile contains water, a coloring matter (biliverdin), cholesterin, saponifiable or saponified fats, chloride of sodium, earthy and alkaline phosphates, carbonates of soda and potass, and a variable quantity of mucus.

The biliary fluid, thus constituted, was for a long time regarded by many as a simple excretion, like the urine; taking no part in digestion, and destined merely to be expelled from the body. It could not, indeed, be shown to exert any such digestive influence on the alimentary substances, as belonged to the gastric and pancreatic juices; and its loss, when excluded from the alimentary canal, did not give rise to any very marked disturbance, certainly not to a suspension of the digestive process. But the experiments of Bidder and Schmidt* seem to have demonstrated conclusively that its presence in the alimentary canal is nevertheless essential to the continuance of life; since animals in which the whole of it is drawn off by a biliary fistula,

---

* Verdauungssaefte und Stoffwechsel. Leipzig, 1852.

though they still feed and digest well, die after a few weeks, reduced to the last degree of debility and emaciation. What the changes are, however, which it undergoes in the intestine, or in what way it is made subservient to the nutritive functions, has never been definitely ascertained.

Bidder and Schmidt have suggested that the organic acids of the biliary salts were probably decomposed in the intestine, as they may be by boiling with caustic potass in a test tube; giving as the result glycine in the one case, and taurine in the other. But neither of these latter substanees have actually been found in the intestine. Liebig again suggested that the bile, or at least its essential ingredients, might be reabsorbed from the alimentary canal, to undergo further modifications elsewhere; and Bidder and Schmidt have shown *(op. cit.)* that the feces of the dog do not contain sulphur enough to account for all the (sulphurous) taurocholic acid which is discharged daily with the bile into the intestine. These facts render it exceedingly probable, if not certain, that the bile is actually reabsorbed, under some form or other, from the alimentary canal; but further than that, there is little or nothing definite, with regard to its physiology, to satisfy the mind of the inquirer. All experimenters, who have undertaken the study of this secretion, have found it the most difficult of investigation of all the intestinal fluids; and yet its importance is so palpable, and its occurrence in different species, orders and classes of animals so universal, that it claims, and must continue to receive the special attention of the physiologist.

Within the past two years we have endeavored to clear up, so far as possible, some of the more obscure points with regard to the history of the bile, and to obtain somewhat more satisfactory notions with regard to its properties and function. The statements which are made in the following pages are derived from the results of sixty-seven different experiments, many of which comprised a series of secondary examinations, and occupied one or two days in their performance. We have examined more particularly the special constitution of the bile in different animals, the best mode of detecting it in intestinal or other fluids, the quantity and time of its discharge into the intestine, its reaction with the gastric and intestinal juices, and lastly its mode of disappearance in the alimentary canal.

*Constitution and Chemical Properties of the Bile.*—The essential ingredients of ox-bile are, as we have mentioned above, two peculiar saline substances, the glykocholate and the taurocholate of soda. They may be obtained in the following manner: The bile is first evaporated to dryness by the water bath. The dry residue is then pulverized and treated with absolute alcohol, in the proportion of at least ℥j of alcohol to every five grains of dry residue. The filtered alcoholic solution has a clear, yellowish color. It contains, beside the glykocholate and taurocholate of soda, the coloring matter and more or less of the fats originally present in the bile. On the addition of a small quantity of ether, a dense, whitish precipitate is formed, which disappears again on agitating and thoroughly mixing the fluids. On the repeated addition of ether, the precipitate again falls down, and when the ether has been added in considerable excess, six to twelve times the volume of the alcoholic solution, the precipitate remains permanent, and the whole mixture is filled with a dense, whitish, opaque deposit, consisting of the glykocholate and taurocholate of soda, thrown down under the form of heavy flakes and granules, part of which subside to the bottom of the test tube, while part remain for a short time in suspension. Gradually these flakes and granules unite with each other and fuse together into clear, brownish-yellow, oily, or resinous looking drops. At the bottom of the test-tube, after two or three hours, there is usually collected a nearly homogeneous layer of this deposit, while the remainder continues to adhere to the sides of the glass in small, circular, transparent drops. The deposit is semi-fluid in consistency, and sticky, like Canada balsam or half-melted resin; and it is on this account that the ingredients composing it have been called the " resinous matters " of the bile. They have, however, no real chemical relation with true resinous bodies, since they both contain nitrogen, and differ from resins also in other important particulars.

At the end of twelve to twenty-four hours the glykocholate of soda begins to crystallize. The crystals radiate from various points in the resinous deposit, and shoot up into the supernatant fluid in white silky bundles. If some of the crystals be removed and examined by the microscope, they are found to be of a very delicate acicular form, running to a finely pointed extremity, and radiating, as already mentioned, in bundles from a central point. As the ether evaporates, the crystals absorb moisture from the air, and melt up rapidly into clear resinons drops; so that it was very difficult to keep them under the microscope long enough for a correct drawing and measurement.

The crystallization in the test tube goes on after the first day, and the crystals increase in quantity for three or four, and even five or six days, until the whole of the glykocholate of soda present has assumed the solid form. The taurocholate, however, is uncrystallizable, and remains in an amorphous condition. If a portion of the deposit be now removed and examined by the microscope, it is seen that the crystals of glykocholate of soda have increased considerably in thickness, so that their transverse diameter may be readily estimated. The uncrystallizable taurocholate appears under the form of circular drops, varying considerably in size, clear, transparent, strongly refractive, and bounded by a dark, well defined outline. *They are not to be distinguished, by any of their optical properties, from oil globules* as they usaally appear under the microscope. They have the same refractive power, the same dark outline and bright centre, and the same degree of consistency. They would be consequently liable at all times to be mistaken for oil globules, were it not for the complete dissimilarity of their chemical properties.

Both the glykocholate and taurocholate of soda are very freely soluble in water. If the mixture of alcohol and ether be poured off and distilled water added, the deposit dissolves rapidly and completely with a more or less distinct yellowish color, according to the proportion of coloring matter originally present in the bile. The two biliary substances present in the solution may be separated from each other by the following means. On the addition of acetate of lead, the glykocholate of soda is decomposed, and precipitates as a glykocholate of lead. The precipitate, separated by filtration from the remaining fluid, is then decomposed in turn by carbonate of soda, and the original glykocholate of soda reproduced. The filtered fluid which remains, and which contains the taurocholate of soda, is then treated with subacetate of lead, which precipitates a taurocholate of lead. This is separated by filtration, and decomposed again by carbonate of soda, as in the former case. The two biliary substances in ox-bile may, therefore, be distinguished by their reactions with the salts of lead. Both are precipitable by the subacetate; but the glykocholate of soda is precipitable also by the acetate, while the taurocholate is not so. If subacetate of lead, therefore, be added to the mixed watery solution of the two substances, and the whole filtered, the subsequent addition of acetate of lead to the filtered fluid will produce no precipitate, because both the biliary matters have been thrown down with the deposit; but, if the acetate of lead be first added, it will precipitate the glykocholate alone, and the taurocholate may afterwards be thrown down separately by the acetate.

*The biliary substances, however, are not the same in different species of animals.* In examining the biliary secretions of different species, Strecker founk so great a resemblanne between them that he was disposed to regard their ingredients as essentially the same. Having established the existence in ox-bile of two peculiar substances, one crystallizable and non-sulphurous (glykocholate), the other uncrystallizable and sulphurous (taurocholate), he was led to consider that the bile in all species of animals as containing the same substances, and as differing only in the relative quantity in which the two were present. The only exception to this was supposed to be pig's bile, in which Strecker found a peculiar organic acid, which he called "hyocholic," or "hyocholinic" acid, in combination with soda as a base.

The above conclusion of his, however, was not entirely correct. The bile of all animals, so far as examined, does, it is true, contain peculiar substances which resemble each other in being freely soluble in water, soluble in absolute alcohol, and insoluble in ether; and in giving also a peculiar reäction with Pettenkofer's test, to be described presently. But, at the same time, these substances present minor differences in different animals, which show them not to be identical.

In dog's bile, for example, there are, as in ox-bile, two substances precipitable by ether from their alcoholic solution; one crystallizable, the other not so. But the former of these crystallizes much more readily than the glykocholate of soda from ox-bile. Dog's bile will not unfrequently begin to crystallize freely in five to six hours after precipitation by ether; while in ox-bile it is usually twelve and often twenty-four and even forty-eight hours before crystillization is fully established. But it is more particularly in their reäction with the salts of lead that the difference between these substances becomes manifest. For, while the crystallizable substance of ox-bile is precipitated by acetate of lead, that of dog's bile is not affected by it. If dog's bile be evaporated to dryness, extracted with absolute alcohol, the alcoholic solution precipitated by ether, and the ether-precipitate then dissolved in water, the addition of acetate of lead to the watery solution produces not the slightest turbidity. If subacetate of lead be then added in excess, a copious precipitate falls, composed of both the crystallizable and uncrystallizble substances. If the lead-precipitate be then separated by filtration, washed and decomposed by carbonate of soda, the watery solution will contain the reformed soda-salts of the bile. The watery solution may then be evaporated to dryness, extracted with absolute alcohol, and the alcoholic solution precipitated by ether, when the ether-precipitate crystallizes partially after a time as in fresh bile. Both the biliary matters of dog's bile are, therefore, precipitable by subacetate of lead, but neither of them by the acetate. Instead of calling them, consequently, glykocholate and taurocholate of soda, we shall speak of them simply as the "crystalline" and "resinous" biliary substances.

In cat's bile the biliary substances act very much as in dog's bile. The ether-precipitate of the alcoholic solution contains here also a crystalline and a resinous substance, both of which are soluble in water, and both precipitable by the subacetate of lead; but neither of them by the acetate.

In pig's bile, on the other hand, there is no crystallizable substance, but the ether-precipitate is altogether resinous in appearance. Notwithstanding this, however, its watery solution precipitates abundantly by both the acetate and subacetate of lead.

In human bile, again, there is no crystallizable substance. We have found that the dried bile, extracted with absolute alcohol, makes a clear, brandy-red solution, which precipitates abundantly with ether in excess; but the ether-precipitate, if allowed to stand, shows no sign of crystallization, even at the end of three weeks. If the resinous precipitate be separated by decantation, and dissolved in water, it precipitates, as in the case of pig's bile, by both acetate and subacetate of lead. This might, perhaps, be attributed to the presence of two different substances, as in ox-bile, one precipitated by the acetate, the other by the subacetate of lead. Such, however, is not the case. For if the watery solution be precipitated by the acetate of lead and then filtered, the filtered fluid gives no precipitate afterward by the subacetate; and if first precipitated by the subacetate, it gives no precipitate, after filtration, by the acetate.

Different kinds of bile vary also in other respects, as, for example, their specific gravity, the depth and tinge of color, the quantity of fat which they contain, &c., &c. Pig's bile is of a nearly clear yellow color, human bile of a dark golden brown, ox-bile of a greenish yellow, dog's bile of a deep brown. The alcoholic solution of dried ox-bile does not precipitate at all on the addition of water, while that of human bile, pig's bile, and dog's bile, precipitates abundantly with distilled water, owing to the quantity of fat which it holds in solution. We have found the specific gravity of pig's bile to be 1030 to 1036; that of human bile 1018; that of ox-bile 1024. These variations, however, are of secondary importance in comparison with those which have been already mentioned, and which show that the crystalline and resinous substances in different kinds of bile, though resembling each other in very many respects, are yet in reality by no means identical.

*Tests for Bile.*—In investigating the physiology of any animal fluid, it is, of course, of the first importance to have a convenient and reliable test by which its presence may be detected. The only test which was for a long time employed in the case of the bile was that which depended *on a change of color produced by oxidizing substances.* If the bile, for example, or a mixture containing bile, be exposed in an open glass vessel for a few hours, the upper layers of the fluid, which are in contact with the atmosphere, gradually assume a greenish tinge, which becomes deeper with the length of time which elapses, and the quantity of bile existing in the fluid. Nitric acid, added to a mixture of bile and shaken up, produces a dense precipitate which takes a bright grass-green hue. Tincture of iodine produces the same change of color, when added in small quantity; and probably there are various other substances which would have the same effect. It is by this test that the bile has so often been recognized in the urine, serous effusions, the solid tissues,

&c., in cases of jaundice. But it is a very insufficient one for anything like accurate investigation, since the appearances are produced simply by the action of an oxidizing agent an the coloring matter of the bile. A green color produced by nitric acid does not, therefore, indicate the presence of the biliary substances proper, but only of the biliverdin. On the other hand, if the coloring matter be absent, the biliary substances themselves cannot be detected by it. For if the biliary substances of dog's bile be precipitated by ether from an alcoholic solution, dissolved in water and decolorized by animal charcoal, the colorless watery solution gives no green color on the addition of nitric acid or tincture of iodine, though it precipitates abundantly by subacetate of lead, and gives the other reäctions of the crystalline and resinous biliary matters in a perfectly distinct manner.

*Pettenkofer's Test.*—This is undoubtedly the best test yet proposed for the detection of the biliary substances. It consists in mixing with a watery solution of the bile, or of the biliary substances, a little cane sugar, and then adding sulphuric acid to the mixture until a red, lake, or purple color is produced. A solution may be made of cane sugar, in the proportion of one part sugar to four parts water, and kept for use. One drop of this solution is mixed with the suspected fluid, and the sulphuric acid then immediately added. On first dropping in the sulphuric acid a whitish precipitate falls, which is abundant in the case of ox-bile, less so in that of the dog. This precipitate redissolves in a slight excess of sulphuric acid, which should then continue to be added until the mixture assumes a somewhat syrupy consistency and an opalescent look, owing to the development of minute bubbles of air. A red color then begins to show itself at the bottom of the test tube, where the drops of sulphuric acid accumulate, which disappears, however, on agitating the mixture. On continuing the addition of sulphuric acid, the red color returns and becomes general, till the whole fluid is of a clear bright cherry-red. This gradually changes to a lake color, and finally to a deep, rich, opaque purple. If three or four volumes of water be then added to the mixture, a copious precipitate falls down, and the color is destroyed.

Various circumstances modify to some extent the rapidity and distinctness with which the above changes are produced. If the biliary substances be present in large quantity, and nearly pure, the red color shows itself at once after adding an equal volume of sulphuric acid, and almost immediately passes into a strong purple. If they be scanty, on the other hand, the red color may not show itself for seven or eight minutes, nor the purple under twenty or twenty-five minutes. If foreign matters, again, not of a biliary nature, be also present, they are apt to be acted upon by the sulphuric acid, and by becoming discolored interfere with the clearness and brilliancy of the tinges produced. On this account it is indispensable, in delicate examinations, to evaporate the suspected fluid to dryness, extract the dry residue with absolvte alcohol, precipitate the alcoholic solution with ether, and dissolve the ether-precipitate in water before applying the test. In this manner all foreign substances likely to do harm will be eliminated, and the test will succeed without difficulty.

It must not be forgotten, beside, that the sugar itself is liable to be acted on and discolored by sulphuric acid when added in excess, and may therefore by itself give rise to confusion. A little care and practice, however, will enable the experimenter to avoid any chance of deception from this source.

When sulphuric acid is mixed with a watery solution containing cane sugar, after it has been added in considerable excess, a yellowish color begins to show itself, owing to the commencing decomposition of the sugar. This color gradually deepens until it has become a dark, dingy, muddy brown; but there is never at any time clear red or purple color unless biliary matters be present. If the bile be present in small quantity, the colors produced by it may be modified and obscured by the dingy yellow and brown of the sugar; but even this difficulty may be avoided by paying attention to the following precautions. In the first place, only very little sugar should be added to the suspected fluid. In the second place, the sulphuric acid should be added very gradually, and the mixture closely watched to detect the first changes of color. If bile be present, the red color peculiar to it is always produced before the yellowish tinge which indicates the decomposition of the sugar. When the biliary matters, therefore, are present in small quantity, the addition of sulphuric acid should be stopped at that point, and the colors, though faint, will then remain clear, and give unmistakable evidence of the presence of bile.

The red color alone is not sufficient as an indication of bile. It is, in fact, only the commencement of the change which indicates the biliary matters. If these matters be present, the color passes, as we have already mentioned, first into a lake, then into a purple; and it is this lake and purple color alone which can be regarded as really characteristic of the biliary reäction.

Pettenkofer has given directions, as quoted by Lehmann, that the elevation of temperature in this experiment, naturally produced by mixing sulphuric acid and water, should not be allowed to exceed 120° F. This, however, is not by any means indispensable. We have often found the lake and purple colors to be produced with the greatest intensity, without taking any precaution to keep down the temperature, and while the test tube was still very hot. Used in this way, Pettenkofer's test may be regarded as of very valuable assistance in the detection of the bile. Its reäction takes place with the bile of all the different species of animals, so far as examined, and with a nearly uniform degree of intensity. It is much more certain and characteristic, therefore, than the test by nitric acid, or tincture of iodine.

*Pettenkofer's reäction is produced by the presence of both, or either of the two biliary substances, crystalline or resinous, and is not dependent on the coloring matter of the bile.*

1. The bile is evaporated to dryness, the dry residue extracted by absolute alcohol, and the alcoholic solution precipitated by ether. The mixture is then allowed to stand until the crystalline and resinous substances have both completely separated. The mixed alcohol and ether are then poured off, and the precipitate dissolved in distilled water and decolorized. The watery solution now gives Pettenkofer's reäction perfectly, though, as previously mentioned, it does not produce any green color with nitric acid and a tincture of iodine.

2. The ether-precipitate of the alcoholic solution of dried ox-bile is dissolved in distilled water. The glykocholate of soda is then precipitated from its watery solution by acetate of lead, separated by filtration, washed, re-composed by carbonate of soda, and again dissolved in water. The remainder of the filtered fluid is then precipitated by subacetate of lead, and the precipitate treated as before with carbonate of soda, and dissolved. The two watery solutions, one containing the glykocholate, the other the taurocholate of soda, both give Pettenkofer's reäction decisively and completely.

Various objections have been urged against this test. It has been stated to be uncertain and variable in its action. Robin and Verdeil (*Chimie Anatomiuue et Physiologique*,) say that its reäctions "do not belong exclusively to the bile, and may, therefore, give rise to mistakes." Some fatty substances and volatile oils (olein, oleic acid, oil of turpentine, oil of caraway) have been stated to produce similar red and violet colors when treated with sugar and sulphuric acid. These objections, however, have not much, if any, practical weight. The test no doubt requires some care and practice in its application, as we have already pointed out; but that is the case, to a greater or less extent, with nearly all chemical tests, particularly those for organic substances. No other substance is liable to be met with in the intestinal fluids of the blood, which would simulate the reäctions of the biliary matters. We have found that the fatty matters of the chyle, taken from the thoracic duct, when tried with Pettenkofer's test, do not give any coloration which would be mistaken for that of the bile. When the volatile oils (caraway and turpentine) are acted on by sulphuric acid, a red color is produced, which afterwards becomes brown and blackish, and a peculiar, tarry, empyreumatic odor is developed at the same time; but we do not get the lake and purple colors spoken of as above. Finally, if the precaution be observed of first extracting the suspected matters with absolute alcohol, then precipitating with ether, and dissolving the precipitate in water, no ambiguity could result from the presence of any of the above substances. The imperfection of the test does not, in fact, consist in its liability to cause other substances to be mistaken for the biliary matters, but in failing sometimes to detect small quantities of the latter when they are really present.

*Pettenkofer's test is not a very delicate one.*

If two drops of dog's bile be added to ʒj of distilled water, and the mixture tried with Pettenkofer's test, it becomes deep cherry red in half a minute, lake in one minute, and a distinct opaque purple in four minutes.

One drop of dog's bile mixed with ʒj distilled water, tried by the same test, becomes cherry red in two minutes and a half, and lake in four minutes; but there is no purple color, even at the end of an hour.

One half a drop of dog's bile with ʒj distilled water, becomes, on the application of Pettenkofer's test, of a somewhat dingy cherry red within a minute; but there is no lake or purple at the end of an hour.

One quarter of a drop of dog's bile with ʒj distilled water, on the addition of sugar and sulphuric acid, becomes immediately yellowish; but afterward only turns of a dingy yellowish brown, hardly, if at all, distinguishable from that produced with a simple solution of cane sugar in water.

Pettenkofer's test, therefore, cannot be relied on for the detection of very minute quantities of the biliary substances. Still it is the best we have, and an admirable one so far as it goes. All chemical tests are limited in this way, with respect to the delicacy of their application. Even Trommer's test for sugar acts very imperfectly with a solution of one-sixteenth of a drop of honey to the drachm of water; and with a solution of one-thirty-second of a drop to the drachm, fails altogether to detect the presence of sugar. Pettenkofer's test, then, if used with care, is extremely useful, and may lead to many valuable results.

With regard to the physiology of the bile, one of the first points which we have endeavored to examine, is the following: *At what period, and how constantly, is the bile discharged into the intestinal canal?* The experiments for this purpose were performed on dogs. The animals were kept confined, and killed at various periods after feeding, sometimes by the inoculation of woorara, sometimes by hydrocyanic acid, but most frequently by section of the medulla oblongata. The contents of the intestine were then collected and examined. In all instances the bile was also taken from the gall-bladder, and treated in the same way with the contents of the intestine, for purposes of comparison. The intestinal contents always presented some differences of appearance when treated with alcohol and ether, owing, probably, to the presence of other substances than the bile; but they always gave evidence of the presence of biliary matters as well. The biliary substances could almost always be recognized under the microscope in the ether-precipitate of the alcoholic solution; the resinous substance under the form of rounded, oily-looking drops, and the other under the form of crystalline groups, generally presenting the appearance of double bundles of slender, radiating, slightly curved or wavy needle-shaped crystals. These substances, dissolved in water, gave a purple color with sugar and sulphuric acid. These experiments were tried after the animals had been kept for one, two, three, five, six, seven, eight, and twelve days without food. The result showed that in all these instances bile was present in the small intestine. It is plainly, therefore, not an intermittent secretion, nor one which is concerned exclusively in the digestive process; but its secretion is constant and it continues to be discharged into the intestine for many days at least after the animal has been deprived of food.

The next point of importance to be examined relates to the *time, after feeding, at which the bile passes into the intestine in the greatest abundance.* Bidder and Schmidt (*op. cit.*) have already investigated this point in the following manner. They operated by tying the common bile-duct and then opening the fundus of the gall bladder so as to produce a biliary fistula by which the whole of the bile was drawn off. By doing this operation, and collecting and weighing the fluid discharged at different periods, they came to the conclusion that the flow of bile began to increase within two and a half to three hours after the introduction of food into the stomach; but that it did not reach its maximum of activity till the end of twelve or fifteen hours. Other observers, however, have obtained different results. Arnold, for example (in *Am. Jour. Med. Sci.*, April, 1856), found the quantity to be largest soon after meals, decreasing again after the fourth hour. Kölliker and Müller, again (in *Am. Journ. Med. Sci.*, April, 1857), found it largest between the sixth and eighth hours. Bidder and Schmidt's experiments, indeed, strictly speaking, show only the time at which the bile is most actively secreted by the liver, but not when it is actually discharged into the intestine. Our own experiments, bearing upon this point, were performed on dogs, by making a permanent duodenal fistula on the same plan that gastric fistulæ have so often been established for the examination of the gastric juice. An incision was made through the abdominal walls, a short distance to the right of the medium line, the floating portion of the duodenum drawn up towards the external wound, opened by a longitudinal incision, and a silver tube, armed at each end with a narrow projecting collar or flange, introduced into it by one extremity, five and a half inches below the pylorus, and two and a half inches below the orifice of the lower pancreatic duct. The other extremity of the tube was left projecting from the external opening in the abdominal patrietes, the parts secured by sutures, and the wound allowed to heal. After cicatrization was complete, and the animal had entirely recovered his healthy condition and appetite, the intestinal fluids were drawn off at various intervals after feeding, and their contents examined. This operation, which is rather more difficult than that of making a permanent gastric fistula, is nevertheless exceedingly useful when it succeeds, since it enables us to study not only the time and rate of the biliary discharge, but also many other extremely interesting matters connected with intestinal digestion. Of five animals operated on, we lost three, and succeeded in retaining a permanent fistula in two.

The results obtained from the experiments on these animals, may be summarily stated as follows. Twenty-four hours after feeding there flows from the fistula a small quantity of fluid, partly brownish and bilious-looking, partly colorless, nearly clear, and more or less frothy. These fluids are mostly neutral, or faintly alkaline, but sometimes have a slightly acid reaction. They come in starts and gushes, sometimes mixed, but very frequently alternating with each other. If the animal be then fed with a full meal (two pounds) of fresh lean meat, during the first fifteen minutes afterward a large quantity of nearly pure bile is poured into the intestine, mingled during the latter part of the time with some gastric juice from the stomach, containing a little albuminose in solution, which precipitates with the bile, forming an opaque bright yellow mixture. This second fluid soon becomes more abundant in proportion to the bile, precipitating a molecular sediment, and becoming less and less strongly colored. In half an hour to an hour, a fine debris of broken-up muscular fibres begins to pass out of the stomach into the intestine suspended in the gastric juice, forming a grayish, gruelly, fluid mixture, in which the proportion of bile to the other ingredients gradually diminishes. This continues from the second to about the twelfth hour, the proportion of muscular debris growing constantly greater, and that of fluid less, so that the mixture is considerably thicker, and more gruelly than a first. The entire quantity of the mixture, also, grows pretty constantly less until the twelfth hour. After that time, the mixture of muscular debris and gastric juice ceases more or less promptly, and the bile becomes again more abundant in proportion to the other ingredients. It is still mixed, however, with the intestinal fluids, and so continues till the end of the twenty-four hours.

In order to ascertain the absolute quantity of bile discharged into the intestine, and its variations during digestion, the duodenal fluids were drawn off, for fifteen minutes at a time, at various periods after feeding, collected, weighed, and examined separately, as follows: each separate quantity was evaporated to dryness, its dry residue extracted with absolute alcohol, the alcoholic solution precipitated with ether, and the ether-precipitate, regarded as representing the amount of biliary matters present, dried, weighed, and then treated with Pettenkofer's test in order to determine, as nearly as possible, their degree of purity or admixture. The result of these experiments is given in the following table. At the eighteenth hour so small a quantity of fluid was obtained that the amount of its biliary ingredients was not ascertained. It reäcted perfectly, however, with Pettenkofer's test, showing that bile was really present.

| Time after feeding. | Quantity of fluid in fifteen minutes. | Dry residue of same. | Quantity of biliary matters. | Proportion of biliary matters to dry residue. |
|---|---|---|---|---|
| Immediately . . | 640 grs. | 33 grs. | 10 grs | .30 |
| 1 hour . . . | 1,990 " | 105 " | 4 " | .03 |
| 3 hours . . | 780 " | 60 " | 4 " | .07 |
| 6 hours . . | 750 " | 73 " | 3½" | .05 |
| 9 hours . . | 860 " | 78 " | 4½" | .06 |
| 12 hours . . | 325 " | 23 " | 3¾" | .16 |
| 15 hours . . | 347 " | 18 " | 4 " | .22 |
| 18 hours . . | — | — | — | — |
| 21 hours . . | 384 " | 11 " | 1 " | .09 |
| 24 hours . . | 163 " | 9½" | 4¼" | .34 |
| 25 hours . . | 151 " | 5 " | 3 " | .60 |

From this it appears that the bile passes into the intestine in by far the largest quantity immediately after feeding and within the first hour. After that time its discharge remains pretty constant, not varying much from four grains (solid biliary matters) every fifteen minutes, or sixteen grains per hour. (This animal weighed 36½ pounds.)

There is, however, an interval, from about the eighteenth to the twenty-first hour, during which its quantity is much less, and the intestine appears to be in a state of temporary inactivity. But though the absolute quantity of bile remains, with this exception, nearly uniform with the above period, its relative quantity to that of the other ingredients increases pretty constantly after the first hour, owing to the diminishing proportion of the digestive fluids and of the alimentary matters which are undergoing solution.

These facts lead us to the consideration of another question which is of great interest in this connection, viz., *what part, if any, does the bile take in digestion?* We have seen that not only its secretion, but also its discharge into the intestine, are both nearly constant, even after the animal has been many days without food; and consequently that it cannot have, like the gastric and pancreatic juices, an exclusive relation to the digestive process. Still, it is actually present also in the intestine during digestion; and is even discharged into it most abundantly immediately after the introduction of food into the stomach, and before the mixture of gastric juice and half-digested food has begun to pass through the pylorus into the intestine. Is this merely a coincidence, or does it have some definite relation to the changes which the food or the bile or both are to undergo in the alimentary canal? With regard to this question the following facts are of some interest.

*The bile precipitates with the gastric juice.*

This precipitation can be readily seen in the fluids drawn from the duodenal fistula, half an hour or more after feeding; when the fluids come, as mentioned above, in intermitting starts and gushes, sometimes neutral or alkaline, clear, and brownish, like nearly pure bile, then as a bright, yellowish, turbid mixture, then nearly colorless and acid, consisting mostly of gastric juice with muscular debris. It can also be seen by mixing in a test tube gastric juice from the dog, obtained by means of a gastric fistula, with bile from the gall-bladder of the same animal. If four drops of bile be added in this way to ℥j of gastric juice, a precipitate falls which contains the whole of the coloring matter of the bile; and if the mixture be then filtered, the filtered fluid passes through quite colorless, and is no longer turned green by nitric acid. The gastric juice, notwithstanding this precipitation, retains its acid reaction, but at the same time loses its power of dissolving albuminous substances. For, if gastric juice which has been precipitated in the above manner be filtered, and then kept in a test tube at the temperature of 100° F. with a piece of boiled white of egg, the white of egg becomes somewhat more transparent and brittle and grows contracted and cracked, but the gastric juice exerts little or no dissolving action upon it. This reaction of bile and gastric juice deserves a closer examination, since the two fluids certainly do mix in the intestine, and must exert a more or less important action on each other.

It is the *biliary substances themselves which cause the precipitation with gastric juice.*

If the crystalline and resinous substances of dog's bile be separated from the other ingredients by the process already several times described, and after precipitation by ether, dissolved in distilled water, they make a clear, colourless solution. Such a solution, made in the proportion of three grain, biliary matters to ℨj of water, precipitates with gastric juice like the bile from the gall-bladder; only the precipitate in this instance is colorless. When fresh bile, therefore, is used, the colouring matter is merely entangled and thrown down with some other substance; the precipitate takes place, however, with the biliary matters proper just as well when the coloring matter is absent.

But *the biliary substances themselves are precipitated;* for if ℥j of filtered gastric juice, taken from the stomach six hours after feeding, be precipitated by the addition of four drops, two drops, or even one drop of bile from the gall-bladder, and the turbid mixture filtered, the clear filtered fluid in every instance gives abundant evidence of the presence of biliary matters by Pettenkofer's test. The biliary substances, therefore, or at least by far the greater part of them, remain in solution and do not fall down with the precipitate by gastric juice.

This reäction of the biliary and gastric fluids, though very important in respect to the theory of intestinal digestion, does not appear to have any particular bearing on the subsequent history of the bile itself. It is the gastric juice and the alimentary substances which are affected by coming in contact with the bile, not the biliary ingredients themselves. The effect of this precipitation, however, on the gastric juice and food, even, is not very thoroughly understood. It has been thought by Bernard that the contact of the bile stops altogether the digestive action of the gastric juice, precipitating at the same time all the albuminose which it had previously dissolved; this precipitated albuminose being afterward dissolved by the action of the pancreatic juice, which is regarded by M. Bernard as an exceedingly active agent in intestinal digestion. Some facts which have been mentioned above, seem, indeed, to support this opinion; as, for example, that the gastric juice, when precipitated with bile, loses its power of artificially dissolving boiled white of egg in a test tube. But it very soon becomes evident to the experimenter that these artificial digestions do not always represent exactly the process as it takes place in the living animal. They may be of great service in suggesting and directing subsequent examinations, but can rarely be depended on exclusively as settling any given question. The gastric fluids, taken from the stomach some time after feeding and filtered, often contain organic matter in solution under a different form from that which it assumes when the digestion is conducted artificially in test tubes. We have found, in point of fact, that bile from the dog's gall bladder always precipitates with the gastric juice as it passes from the stomach into the intestine, whether it be taken from the stomach fifteen minutes, half an hour, or six hours after feeding. The acid fluids drawn from the duodenal fistula, also, three and six hours after feeding, will precipitate with those drawn twenty and twenty-five minutes after feeding, and in which the proportion of biliary matters is larger.

But there are some considerations which militate against the simple view of intestinal digestion entertained by M. Bernard. In the first place the gastric fluids precipitate with the pancreatic juice as with the bile, when mixed in a test tube; while the two intestinal fluids, bile and pancreatic juice, do not precipitate with each other. In the second place, it is a remarkable fact that bile very constantly *finds its way into the stomach, in larger or smaller quantities, at almost all periods of digestion.* This fact has already been noticed by Lehmann, who states (*Physiological Chemistry*, Philad. ed.. vol i. p. 447) that he has "made few examinations of human bodies, or even of recently killed healthy animals, in which he has not discovered biliary constituents in the contents of the stomach lying near the pyloric end." In the pig it is almost universal to find the pyloric portion of the gastric mucous membrane after death pesmanently stained of a bright yellow by bile. It is very common indeed, furthermore, while drawing off the fluids of the stomach by a gastric fistula within the first fifteen minutes after feeding, to see bile suddenly make its appearance, evidently by regurgitating through the pylorus, when the clear, colourless gastric juice instantly becomes turbid and yellowish. In a few moments the bile may cease to present itself, and the gastric fluids regain their colourless and transparent appearance, to be again, perhaps, rendered turbid and yellow some moments afterward. The gastric fluids, even, which are drawn six hours after feeding, and which are thick, grayish and gruelly in appearance, if filtered clear, will frequently give distinct evidence of the presence of bile by Pettenkofer's test. All this certainly does not essentially interfere with the process of gastric digestion; and the action of the bile on the digested food in the intestine evidently does not correspond exactly with any explanation which has been suggested.

The next series of experiments to which we resorted were undertaken in order to investigate, so far as possible, the followin very obscure question, viz: *What becomes of the bile in its passage through the intestine?* The dogs used for these experiments were fed with fresh meat and then killed at various intervals after the meals, the abdomen opened, ligatures placed upon the intestines at different points, and the contents of their upper, middle and lower portions collected and examined separately. The results thus obtained show that, under ordinary circumstances, the bile, which is quite abundant in the duodenum and upper part of the small intestine, diminishes in quantity from above downward, and is not to be found in the large intestine. The entire quantity of the intestinal contents diminishes and their consistency increases as we approach the ileo-cæcal valve. At the same time their colour changes from a light yellow to a dark bronze, or blackish green, which is always strongly pronounced in the last quarter of the small intestine. The following experiment will serve to show the plan which was followed and its results.

*Experiment.*—A full-grown, healthy bitch, was fed with fresh meat, then kept entirely without food, but supplied only with water, for five days. At the end of that time she was killed by section of the medulla.

The stomach contained fl℥ss of a nearly colorless, dingy, frothy, neutral fluid.

Upper half of small intestine contained 117 grains of a dull yellow, gelatinous, semi-fluid, neutral matter. Its dry residue was 24 grains.

Lower half of small intestine contained 130 grains of a much darker bronze-colored neutral gelatinous mass, a large proportion of which consisted of hairs and intestinal worms, which were not present in the contents of upper half. Dry residue 20 grains.

Large intestine contained 210 grains of a dark, olive-brown, consistent mass, slightly acid in reaction. Dry residue 73 grains.

All three lots were then evaporated to dryness, and afterward exhausted by repeated extraction with absolute alcohol, until the filtered alcohol came through perfectly colorless, and no longer gave any turbidity with ether. The alcoholic solutions were then reduced by evaporation to the same volume and precipitated with ether in excess. The ether-precipitate was separated, dried under the air-pump, and weighed. The results obtained in this way are given in the two following tables:

Table 1.

| | Weight of fresh contents. | Dry residue of same. | Ether-precipitate. | Proportion of ether precip. to fresh contents. |
|---|---|---|---|---|
| Upper half of small intestine | 117 grains. | 24 grains. | 5 grains. | .0427 |
| Lower " " | 130 " | 20 " | 5 " | .0384 |
| Large intestine . . . . | 210 " | 73 " | 3 " | .0142 |

Table 2.

| | Weight of solid residue. | Ether-precipitate. | Prop'n of ether-precip. to solid residue. |
|---|---|---|---|
| Entire small intestine . . | 44 grains. | 10 grains. | .227 |
| Large intestine . . . . | 73 " | 3 " | .041 |

The ether-precipitate of the alcoholic solution is, therefore, both positively and relatively very much less in the large intestine than in the small. Its proportion to the entire solid contents is only one-fifth or one-sixth as great in the large as it is in the small intestine. But even this small quantity does not consist of biliary matters; for the dried ether precipitates, in the above experiment, when dissolved in distilled water all three precipitated by subacetate of lead; but that from the large intestine very much the least. The watery solutions being treated with sugar and sulphuric acid, those from both portions of the small intestine gave Pettenkofer's reäction promptly and perfectly in less than a minute and a half; while in that from the large intestine no red or purple color was produced even at the end of three hours, but only a dingy, muddy brown.

The small intestine, consequently, contains at all times substances giving all the reactions of the biliary ingredients; while in the contents of the large intestine no such substances can be recognized by Pettenkofer's test.

The biliary matters, therefore, disappear in their passage through the intestine. This disappearance must be explained in two different ways. First, the biliary matters may be actually reäbsorbed from the intestine, and taken up by the bloodvessels; or second, they may become so altered and decomposed by the intestinal fluids as to lose the power of giving Pettenkofer's reäction with sugar and sulphuric acid, and pass off with the feces in an insoluble form. The first of these explanations is that which has recently been regarded with the most favor; and it is, in fact, rendered extremely probable by the experiments of Bidder and Schmidt, already referred to, in which they found that the entire quantity of sulphur contained in the feces of the dog during five days was very much less than that which must have been discharged with the bile into the intestine during the same time. It is almost impossible to avoid the conclusion that it has been reabsorbed by the bloodvessels. Still this gives us no idea how far the bile is altered before its reäbsorption; and the direct and absolute proof, also, of finding the biliary matters in the blood of the portal vein is still wanting. We have endeavored to supply the latter deficiency by examining the portal blood in dogs, killed at various times after feeding. The animals were killed by section of the medulla oblongata, a ligature immediately placed on the portal vein, while the circulation was still active, and the requisite quantity of blood collected. This blood was sometimes immediately evaporated to dryness by the water bath. Sometimes it was coagulated by boiling in a porcelain capsule over a spirit lamp, with water and an excess of sulphate of soda, and the filtered watery solution afterward examined. But most frequently, the blood, after being collected from the vein, was coagulated by the gradual addition of three times its volume of alcohol at ninety-five per cent., stirring the mixture constantly, so as to make the coagulation gradual and uniform. It was then filtered, the moist mass remaining on the filter subjected to strong pressure in a linen bag by a porcelain press, and the fluid thus obtained added to that previously filtered. The entire spiritous solution was then evaporated to dryness, the dry residue extracted with absolute alcohol, and the alcoholic solution treated as usual to discover the presence of biliary matters. In every instance blood was taken at the same time from the jugulars or the abdominal vena cava, and treated in the same way for the pnrpose of comparison. We have examined the blood in this way one, four, six, nine, eleven and a half, twelve and twenty-four hours after feeding. As the result of these examinations it was found that in the venous blood, both of the portal vein and of the general circulation, there exists a substance soluble in water and absolute alcohol, and precipitable by ether from its alcoholic solution. This substance is often considerably more abundant in the portal blood than in that from the general system. It adheres closely to the sides of the glass after precipitation, so that it is always difficult and often impossible to obtain enough of it mixed with ether for microscopic examination. It dissolves also, like the biliary substances, with great readiness in water; but in no instance have we ever been able to obtain from it such a satisfactory reaetion of Pettenkofer's test, as would indicate the presence of bile. This is not because the reaction is masked, as might be suspected, by some of the other ingredients of the blood; for if at the same time two drops of bile be added to half an ounce of blood taken from the abdominal vena cava, and the two specimens treated alike, the ether precipitate may be considerably more abundant in the case of the portal blood will give no such reaction. Notwithstanding, then, the strong probability that the biliary matters are taken up by the portal blood, we have failed to recognize them there by Pettenkofer's test.

From the facts, therefore, which have been detailed above, we may derive the following conclusions:

I. The two biliary substances, crystalline and resinous, are not the same in different species of animals, though they resemble each other in most of their chemical properties.

II. In all cases, they act in the same way with Pettenkofer's test, whether both or only one of them be present.

III. In different kinds of bile, the biliary matters are to be distinguished from each other principally by their reäction with the salts of lead.

IV. In human bile there is no crystallizable biliary substance, but only a resinous one. The same thing is the case with the bile of the pig.

V. Pettenkofer's reaction is the only available test for the biliary substances proper. It may fail to detect them when present in very small quantity; but, if used with care, will not lead us to mistake other substances for them.

VI. The bile in the carnivorous animals passes into the intestine for at least twelve days after the last meal.

VII. It is discharged into the intestine most abundantly immediately after feeding; during the remainder of the twenty-four hours its flow is about uniform (sixteen grains biliary matters per hour in a medium sized dog), except from about the eighteenth to the twenty-first hour, during which time it is much less.

VIII. When the bile comes in contact with the gastric fluids, the organic matters of the latter are precipitated; but the biliary substances remain in solution.

IX. The biliary substances disappear during their passage through the intestine, so that they can no longer be recognized by Pettenkofer's test.

X. They are, in all probability, reäbsorbed into the blood; but if so, they first undergo in the intestine such changes, that they no longer give Pettenkofer's reaction with sugar and sulphuric acid.

---

*Gonorrhœa.*—Mr. Dallas of Odessa (Med. Chir. Rev. July, 1856) confirms the statements of Taddei, Marchal and others, that copaiba injections afford the most efficacious treatment of gonorrhœa. He reports sixteen cases cured, without internal remedies, by repeated injections of the following mixture: Copaibæ, drach. 5; vitell. ovi unius; ext. opii, gr. j; aquæ, oz. vij. Dr. Henry Hancox (Lancet, August 1856) pronounces buchu as effectual as copaiba in the treatment of gonorrhœa.

